# Evaluation of the simulation based training quality assurance tool (SBT-QA10) as a measure of learners’ perceptions during the action phase of simulation

**DOI:** 10.1186/s12909-023-04273-6

**Published:** 2023-05-01

**Authors:** Kim Ekelund, Stephanie O’Regan, Peter Dieckmann, Doris Østergaard, Leonie Watterson

**Affiliations:** 1 Copenhagen Academy for Medical Education and Simulation, Capital Region of Denmark, Denmark; 2 Faculty of Health and Medical Sciences, Copenhagen University, Copenhagen, Denmark; 3 Sydney Clinical Skills and Simulation Centre, Royal North Shore Hospital, St Leonards, New South Wales Australia

**Keywords:** Simulation-based training, Simulation-based learning, Questionnaire development, Construct validity, Observer role, Quality Assurance Tool, Learners’ perception

## Abstract

**Background:**

In an earlier interview-based study the authors identified that learners experience one or more of eight explicit perceptual responses during the active phase of simulation-based training (SBT) comprising a sense: *of belonging to instructor and group, of being under surveillance, of having autonomy and responsibility for patient management, of realism, of an understanding of the scenario in context, of conscious mental effort, of control of attention*, and *of engagement with task*. These were adapted into a ten-item questionnaire: the Simulation Based Training Quality Assurance Tool (SBT-QA10) to allow monitoring of modifiable factors that may impact upon learners’ experiences. This study assessed the construct validity evidence of the interpretation of the results when using SBT-QAT10.

**Materials and methods:**

Recently graduated doctors and nurses participating in a SBT course on the topic of the deteriorating patient completed the SBT-QAT10 immediately following their participation in the scenarios. The primary outcome measure was internal consistency of the questionnaire items and their correlation to learners’ satisfaction scores. A secondary outcome measure compared the impact of allocation to active versus observer role.

**Results:**

A total of 349 questionnaires were returned by 96 course learners. The median of the total score for the ten perception items (TPS) was 39 (out of 50), with no significant difference between the scenarios. We identified fair and positive correlations between nine of the 10 items and the SBT-QA10-TPS, the exception being “mental effort”. Compared to observers, active learners reported significantly more positive perceptions related to belonging to the team and interaction with the instructor, their sense of acting independently, and being focused. The questionnaire items were poorly correlated with the two measures of global satisfaction.

**Conclusion:**

Except for the item for mental effort, the QA10-TPS measures learners’ experiences during the active phase of simulation scenarios that are associated with a positive learning experience. The tool may have utility to learners, instructors, and course providers by informing subsequent debriefing and reflection upon practice for learners and faculty. The relationship between these perceptions and commonly used measures of satisfaction remains poorly understood raising questions about the value of the latter.

**Supplementary Information:**

The online version contains supplementary material available at 10.1186/s12909-023-04273-6.

## Background

The features of simulation-based training (SBT) that contribute to effective learning have been studied extensively. [1–9] SBT typically features three phases: pre-, in- and post-action. The pre-action phase includes introduction to the simulator and the scenario briefing when learners are prepared for the in-action phase, where they actively interact with a simulator and a team to manage a clinical task. Facilitated debriefing conversations are most often conducted in the post-action (post-scenario) phase. All three phases of SBT are assumed to contribute to learning. [2–7, 10, 11] The learners’ real time experiences during the in-action phase of simulation and its impact on their satisfaction or learning outcomes have been investigated in different studies. [12–14] Simulation learners’ physical, psychological, and social perceptions act as fiction and / or reality cues in the in-action experience during scenarios. [15] Recently, we identified that the learners experience one or more of the following eight explicit perceptual responses during the in-action phase of simulation and associated these perceptions with phrases evoking emotions and/or social relationships with others and/or their cognitive understanding of the situation: [16] *1.belonging to instructor and group, 2.of being under surveillance, 3.of having autonomy and responsibility for patient management, 4.a sense of realism, 5.an understanding of the scenario in context, 6.a sense of conscious mental effort, 7.control of attention*, and *8.engagement with task*.

These perceptions appeared to be consistently attributed to learner reported satisfaction with the activity. As an additional observation, learners appeared to associate these perceptions with modifiable aspects of instructional design and instructor behavior, which were termed ‘enablers’ and with factors predating the scenario, including prior experience and expectations of SBT. The authors concluded that it would be valuable to monitor these perceptions during courses for the purposes of intervening during a course if learners report negative perceptions that may impact upon their experience, to feedback to instructors and for quality assurance of instructional design. [16] Subsequently, we converted these perceptions into a ten-item questionnaire: the Simulation Based Training Quality Assurance Tool (SBT-QA10).

Our present study primarily aimed to assess the SBT-QA10’s construct validity in appraising learners’ perceptions during the action phase of SBT for the purpose of informing the debrief and or other aspects of the learning activity and with the broader purpose of informing the design and delivery of future simulation programs.

## Methods

### Overview

The study was approved by the local institutional review board (AU14E0935) and conducted as a single-center prospective single cohort questionnaire study at the Sydney Clinical Skills and Simulation Centre (SCSSC), within the Northern Sydney Local Health District (NSLHD), in Sydney, Australia. Learners completed the SBT-QAT10 at the end of scenarios in standardized SBT course.

Considering the relatively limited intended application of the tool was to be personal reflection and programmatic level quality assurance, we adopted classical notions of criterion validity and construct validity after referring to Messick’s argument-based validity framework [17] where these correspond closely Messick’s dimension of *Relationships with other variables*. Consequently, construct validity evidence was assessed by measuring the internal consistency of the questionnaire items, their correlation with satisfaction scores and the comparative impact of the assignation of active versus observer roles on learners’ perceptions. [18]

We hypothesized that the SBT-QA10’s questionnaire items are internally consistent and that positive perceptions will be associated with positive overall satisfaction scores.

As further confirmation of the construct validity we also sought to appraise the tool against a modifiable determinant of learners’ experience. Emerging evidence suggests one factor impacting upon the learning experience is the learner’s assigned role as either active participant versus observer in the action phase of SBT. [18–20] The learners’ experience when in observer roles is relevant to the instructional design and facilitation of simulation programs. Consequently, we measured the comparative impact of the assignation of active versus observer roles on learners’ perceptions as a secondary measure of the construct validity.

### Learners

Learners were newly graduated nurses or doctors within their first 12 months of postgraduate work who were enrolled in a SBT based course to fulfil mandatory training requirements entitled Detection, Evaluation, Treatment, Escalation and Communication in Teams (DETECT) that addressed management of the deteriorating patient on a hospital ward. The course was similar in format to one utilized in previous studies. [16, 21] The study was performed accordance with the Declaration of Helsinki and approved by the appropriate ethics committee. All learners received written and oral information regarding the study and consented voluntarily to having data used for study purposes. Learners could withdraw their consent at any time.

### Questionnaire development

The SBT-QAT10 questionnaire tool was developed, as an iterative process with the team-members, by adapting definitions of the eight perceptions provided in the original qualitative study into action statements and by incorporating questionnaire items that were used in a previously published evaluation of the DETECT course that addressed learner satisfaction. [16, 21] In the original interview study, the learners used phrasing to describe their perceptions in a manner to suggest two overarching themes: psychosocial emotional responses and cognitive understanding of the situation. Belonging was a perception that was commonly expressed with predominantly psychosocial phrasing, for example. In contrast, perceptions of conscious mental effort, task focus and control of attention were predominantly concerned with the learners’ cognition. Other perceptions, including surveillance, responsibility, realism, and contextual understanding, showed a greater degree of inter-individual variation in that they had a psychosocial impact on some learners and an impact on the cognitive processes of other learners”. [16, 21] We intentionally avoided classifying the questionnaire items into themes to enable further exploration through statistical factor analysis.

We developed the two additional questionnaire items as two of the original perceptions were represented by two separate related factors: The perception ‘*belonging*’ represented both relationships formed within the participant group and between learners and the instructor (Q1-2). [16] Similarly, the perception ‘*responsibility*’ represented both the amount of autonomy and independence available to the participant as well as the level of available support from the faculty member (instructor) (Q4-5). [16] In developing of the SBT-QA10, we tried to avoid survey fatigue by altering use of positive and negative statements in the questionnaire (avoiding “straightening” the answers). [22, 23] The final questionnaire contained a total of 10 items for perceptions of which seven were positively phrased and three were negatively phrased.

Two measures of satisfaction were also included in the final questionnaire. The phrases “I felt comfortable learning this way” and “I now feel more confident managing the clinical case” were derived without changes in wording from the previously cited study where they were shown to discriminate between the modifiable factors of technology format (conventional face to face versus videoconference enabled remote facilitation) and instructor experience with SBT. [21] A five-point Likert scale was used to score level of agreement with the questionnaire item.

The readability of the questionnaire was also tested as part of an iterative process and was piloted by members of a teaching faculty within the SCSSC prior to deployment. [24, 25]

### DETECT course

This course is part of a larger program of emergency response team training and has been described in detail in previous publications. [16, 21] DETECT courses have low complexity scenarios designed to rehearse identifying, communicating, and treating patient deterioration on a hospital ward. The scenarios are designed to be of equal difficulty and matched to the learners’ level of clinical experience and workplace roles. The course is run in a pause-and-discuss format where learners in groups of six to eight engage in one or two phases of action that are interspersed with targeted reflective debriefing conversations.

Following an introductory lecture, learners rotate through three simulation scenarios that employ either patient simulators or simulated patients. To accommodate larger group sizes, a fourth scenario may be conducted in the format of a table-top case scenario, whereby facilitators use a verbal description and visual props to present the scenario. [26]

During each scenario round, the groups are divided into learners undertaking active and observer roles. The learners self-select the roles, with instructors ensuring that all learners experience both roles across the scenario rounds. Learners with active roles are briefed that they would take part in the action phase of the scenario and debriefing conversations. Learners with observer roles are briefed that they would observe the action phase from within the room and contribute their perspective to the debriefing conversations during the pauses.

The course was slightly modified to enable the questionnaires to be completed. This included a preliminary briefing about the research project including providing questionnaire forms and collecting completed consent forms allowing analysis of data. Immediately after the in-action phase, and before the post-scenario debriefing, the learners were asked to fill in the questionnaire (both active and observers). Questionnaire completion was expected to require five minutes. As we did not intend to modify the DETECT course during the study period, the results were not analyzed by the authors until the study period was over.

### Instructors

Each DETECT course is delivered by three to four instructors. Each scenario has one instructor who delivers their scenario three to four times per course, with learners rotating between scenarios. Prior to instructing on the course, every instructor completes the course-specific instructor accreditation program and achieves the course standard for competency in scenario delivery and constructive student enquiry-focused debriefing. [21] The authors were not involved in teaching and recruiting in the included courses to avoid creating any bias.

### Randomization

As DETECT is mandatory training for the organization it was important that the course remained unaffected by the study and therefore no explicit randomization was performed. Learners were divided into equal sized groups by the course leader during the course introduction without any knowledge of the learners’ demographic attributes, apart from gender, profession, and estimated age. No group of learners had identical rotations.

### Outcome measures

#### Primary outcomes

Construct validity was assessed by correlating the scores for the individual SBT-QA10 items with scores for other perceptions, the total score of the ten items and with scores for the two individual measures of satisfaction.

#### Secondary outcomes

Learners’ responses to the SBT-QA10 questionnaire were also compared according to their assigned roles as ‘active responders’ or ‘observers’ according to our hypothesis that the strength of the perception would differ between learners in each group. For instance, in comparison to responses provided by learners in active roles, we predicted that learners in observer roles would report reduced scores for realism and mental workload. We also compared questionnaire responses between the second versus first scenario in a given role. Here, we hypothesized that scores for some perceptions such as being observed, use of mental workload and feeling uncomfortable may change reflecting greater familiarity with the teaching environment and methods, an effect observed in the previous study. [13]

### Statistics

#### Data handling

Questionnaire results were analyzed in Microsoft ® Excel (version 16.23 (190,309)™). Responses for the negatively phrased questionnaire items and the items predicted to have a negative impact on learners’ satisfaction, were reversed so that a high score for each item would be assumed to predict a positive experience. Missing responses for individual questionnaire items or within the set of twelve questionnaire items were treated as missing data and excluded from analysis.

#### Statistical tests

Data were analyzed using IBM SPSS (version 21). Tests requiring two-group comparison of independent and dependent variables used the Kruskal-Wallis non-parametric test for ordinal or categorical data or where interval data were not normally distributed. Tests requiring correlation employed two tailed Kendall’s tau Rank Order Test of Correlation for non-parametric data. A significance value of *p* < 0.05 was accepted as significant for all tests.

An exploratory principal component factor analysis (EPCFA) was conducted to further validate the questionnaire items and explore underlying themes. Generally accepted criteria for EPCFA were applied including an Eigenvalue > 1, factor loading of > 0.4, a Kaiser-Meyer-Olkin (KMO) Measure of Sampling Adequacy > 0.7 and Bartletts Test for Sphericity reaching significance of < 0.05. [27].

The eligible populations of these groups average 200 per annum. Based on previous studies, we assumed 50 learners would provide adequate power at 0.8 to detect 10% difference between the groups with 2-sided significance levels of *p* < 0.05. [8, 28]

## Results

### Study design, setting and learners

A total of 96 learners agreed to participate in the study from a total of 103 eligible participants who attended the DETECT course on one of three dates between November 2018 and January 2019 (Table 1). In total 349 questionnaires were returned of which thirty-nine were removed, due to one or more items being left unanswered. All 96 learners participated in three scenarios, in which the action phase was conducted using a patient simulator or simulated patient, and 61 learners participated in a fourth scenario.

**Table 1:** Baseline Characteristics (*n* = 96)

Learners		
Gender (*n*)	Women	53
	Men	41
	Other	2
Profession (*n*)	Nurses	16
	Doctors	72
	Others*	8
Experience (in years) **	Median (range)	0.0 (0–20)
	Mean	0.84
No of times in simulation before (*n*)	0	19
	1–5	48
	> 5	26
	NA	3

*n*: Number, NA: Not Answered, *Others: 5 nurse assistants, 1 nurse student, 2 NA.

**Ten experienced (3–20 years) doctors participated in DETECT.

### SBT-QA10 questionnaire

The median scores and interquartile range (IQR) after each scenario including the Total Perception Score (SBT-QA10-TPS) were calculated for individual questionnaire items and the two items for comfort and confidence within each round of scenarios and for the total of all scenarios.

The Median (IQR) SBT-QA10-TPS across all scenarios was 39 [5] with no significant differences observed between the scenarios (Kruskal-Wallis test *p* = 0.96). The median (IQR) for the four scenarios 1, 2, 3, and 4 were 39 [5], 40 [6], 40 [5] and 39 [5], respectively. The distributions of the TPSs were negatively skewed for all scenarios, indicating that most of the scores were in the upper half of the scale.

The ten individual SBT-QA10 items for perception were correlated with one another and the SBT-QA10-TPS to determine the extent to which they contributed to positive perceptions. We identified fair correlations between nine of the ten items and the SBT-QA10-TPS, the exception being “mental effort”. [29] Nine items were positively correlated as we had predicted, after inverting scores for negatively phrased items, with “mental effort” showing a poor and negative correlation. These findings support the validity of the use for the above mentioned nine items.

In contrast, we identified poor correlations between the ten items and comfort or confidence. [30] (Table 2).

**Table 2:** Correlations between individual perceptions scores, the satisfaction scores (Comfortable and Confident) and the total perception score (SBT-QA10-TPS).

		Correlations				
Item	Scores	Scen 1 n = 83	Scen 2 n = 83	Scen 3 n = 87	Scen 4 n = 57	All scen n = 310
1. I felt part of the team	Comfortable Confident	**0.2** **0.3**	**0.3** **0.2**	**0.3** **0.3**	**0.3** 0.2	**0.3** **0.3**
	SBT-QA10-TPS	**0.4**	**0.5**	**0.4**	**0.5**	**0.5**
2. The faculty member(s) interacted well with me	Comfortable Confident	0.1 0.1	0.1 0.1	0.1 0.1	**0.3** 0.1	**0.1** 0.1
	SBT-QA10-TPS	**0.5**	**0.5**	**0.6**	**0.5**	**0.5**
3. Being observed did not intimidate me *	Comfortable Confident	0.2 0.2	0.2 0.1	− 0.01 0.1	0.2 **0.3**	**0.1** **0.2**
	SBT-QA10-TPS	0.1	0.3	**0.3**	**0.4**	**0.3**
4. I felt I was able to act as independently as I wanted to	Comfortable Confident	**0.2** **0.2**	**0.4** **0.3**	**0.3** **0.3**	**0.3** 0.01	**0.3** **0.2**
	SBT-QA10-TPS	**0.5**	**0.5**	**0.5**	**0.5**	**0.5**
5. I felt adequately supported by the faculty member(s)	Comfortable Confident	**0.2** 0.1	**0.2** 0.1	**0.3** **0.2**	0.2 0.01	**0.2** **0.1**
	SBT-QA10-TPS	**0.6**	**0.5**	**0.5**	**0.5**	**0.5**
6. I felt that the scenario was realistic *	Comfortable Confident	**0.3** 0.06	.**3** 0.1	0.1 − 0.03	0.2 − 0.005	**0.2** 0.03
	SBT-QA10-TPS	**0.5**	**0.5**	**0.4**	**0.7**	**0.5**
7. I understood the purpose of the scenario	Comfortable Confident	**0.2** 0.2	**0.3** **0.3**	**0.2** 0.2	0.2 − 0.04	**0.3** **0.2**
	SBT-QA10-TPS	**0.6**	**0.4**	**0.4**	**0.5**	**0.5**
8. It did not require a lot of mental effort to play my role in the scenario*	Comfortable Confident	0.000 − 0.06	0.005 − 0.05	− 0.2 0.05	− 0.03 0.06	− 0.05 0.01
	SBT-QA10-TPS	0.1	0.1	0.07	− 0.06	0.07
9. I was not distracted by non-relevant objects and events during the scenario *	Comfortable Confident	0.1 0.2	0.2 0.2	0.1 0.1	0.05 0.2	**0.1** **0.2**
	SBT-QA10-TPS	**0.6**	**0.5**	**0.6**	**0.6**	**0.6**
10. I was focused on being involved in the scenario	Comfortable Confident	**0.4** **0.2**	**0.4** **0.3**	**0.3** 0.2	**0.3** − 0.02	**0.3** **0.2**
	SBT-QA10-TPS	**0.5**	**0.4**	**0.3**	**0.4**	**0.4**
11. I feel comfortable learning this way	SBT-QA10-TPS	**0.3**	**0.4**	**0.2**	**0.3**	**0.3**
12. I feel confident managing a similar clinical case in the future	SBT-QA10-TPS	**0.2**	**0.3**	**0.3**	0.1	**0.2**

* Denotes a question where scores have been inverted. Scen is Scenario

Comfortable: “I feel comfortable learning this way” / Confident:” I feel confident managing a similar clinical case in the future”.

SBT-QA10-TPS: Total perception score of items 1–10.

Correlations are considered as Poor (0-0.2), Fair (0.3-0.5) Moderate (0.6-0.7) Very strong (0.8-0.9) or Perfect (1.0). [30]

Correlations shown in **bold** are significant at the 0.05 level (2-tailed).

Statistical analysis: non-parametric test, Kendall’s Tau B.

When compared to determine possible differences across scenarios eight items demonstrated no significant differences, whereas two items, surveillance, and mental effort, displayed significant trends: Learners reported increasing ranked scores from round 1 to 4 for feeling not intimidated by being observed, i.e., they felt increasingly less intimidated. Learners reported decreasing ranked scores for reporting that it did not require mental effort, i.e., it required increasingly more mental effort as the day progressed. These results only weakly support the use of the tool (data not shown).

An exploratory factor analysis identified four factors which accounted for 62% of the variance. (Table 3). Factor 1 included three items: (I felt part of the team, The faculty interacted well, I felt supported) Factor 2 contained three of the SBT-Q10 items (I acted independently, I understood purpose of scenario, I was focussed) including the two global satisfaction items Factor 3 contained two items related to perceptions of surveillance and realism and the fourth factor contained two perceptions related to mental effort and distraction. Reliability analysis for each factors identified Cronbach’s Alpha of 0.814, 0.667, 0.854, and 0.130, respectively.

**Table 3:** Exploratory factor analysis of the questionnaire items

Rotated Component Matrix^a^				
Items/Component	1	2	3	4
I felt part of the team	0.707			
2. The faculty member(s) interacted well with me	0.864			
3. Being observed did not intimidate me *			0.857	
4. I felt I was able to act as independently as I wanted to		0.497		
5. I felt adequately supported by the faculty member(s)	0.853			
6. I felt that the scenario was realistic *			0.807	
7. I understood the purpose of the scenario		0.445		
8. It did not require a lot of mental effort to play my role in the scenario*				0.779
9. I was not distracted by non-relevant objects and events during the scenario				0.641
*10. I was focused on being involved in the scenario		532		
11. I feel comfortable learning this way		0.774		
12. I feel confident managing a similar case in the future		0.720		

* Denotes a question where scores have been inverted. ^**a**^ Components values < 0.4 are not shown.

Reliability analysis identified Cronbach’s Alpha of 0.814, 0.667, 0.854, and 0.130, respectively.

Learners’ responses to the SBT-QA10 questionnaires were analyzed according to their roles as ‘active responders’ or ‘observers’. We hypothesized that learners in the active group would report more positive scores. For instance, it was expected that the active learners were likely to score higher in realism and focus, feeling part of the team, interacting with the instructor, acting independently, and being observed and have a higher TPS (Table 4). Significant differences were found where active learners reported more positive perceptions related to belonging to the team and interaction with the instructor, their sense of acting independently, and being focused. Active learners reported higher mental effort than observers.

**Table 4:** The Individual Perceptions Scores Presented for Active and Observer Roles for all scenarios *(n* = 253)

Items	Roles	Median (IQR#)
1. I felt part of the team	Active	**4 (1)**
	Observer	**4 (1)**
2. The faculty member(s) interacted well with me	Active	**5 (1)**
	Observer	**4 (1)**
3. Being observed did not intimidate me *	Active	3 (2)
	Observer	3 (1)
4. I felt I was able to act as independently as I wanted to	Active	**4 (1)**
	Observer	**3 (1)**
5. I felt adequately supported by the faculty member(s)	Active	**4 (1)**
	Observer	**4 (1)**
6. I felt that the scenario was realistic *	Active	4 (1)
	Observer	4 (1)
7. I understood the purpose of the scenario	Active	5 (1)
	Observer	4 (1)
8. It did not require a lot of mental effort to play my role in the scenario*	Active	**2.5 (1)**
	Observer	**3 (1)**
9. I was not distracted by non-relevant objects and events during the scenario *	Active	**4 (1)**
	Observer	**4 (2)**
10. I was focused on being involved in the scenario	Active	**4 (1)**
	Observer	**4 (1)**
11. I feel comfortable learning this way	Active	4 (1)
	Observer	4 (0)
12. I feel confident managing a similar clinical case in the future	Active	4 (1)
	Observer	4 (1)
13. SBT-QA10-TPS	Active	**40 (4)**
	Observer	**39 (5)**

# IQR: Inter Quartile Range (IQR = Q3-Q1, where Q1 and Q3 are 25% and 75% quartile, respectively).

* Denotes a question where scores have been inverted.

SBT-QA10-TPS: Total perception score of items 1–10.

Medians shown in **bold** are significant different at the 0.05 level (2-tailed).

Statistical analysis: non-parametric test, Kruskal-Wallis rank sum test.

For completeness, we also analyzed the influence of gender, profession, and previous exposure to SBT (data not shown). Only learners identifying as “Female” or “Male” were included in the in these statistical calculations. Learners identifying as “Other” (*n* = 2) were omitted here as numbers were too small. No significant gender differences were observed in total SBT-QA10-TPS. Female learners reported lower scores for surveillance suggesting they found it more intimating to be observed but were more comfortable and confident learning by SBT compared to their male colleagues. No significant differences were observed in the total SBT-QA10-TPS for profession. According to the SBT-QA10 responses, nurses felt significantly more as part of a team, more observed but also more supported than doctors. Compared with nurses, doctors reported feeling less confident to manage similar clinical case in the future. When focusing on the learners’ simulation experience: Novices felt that the scenarios were more unrealistic, they felt challenged to follow the scenarios, but were despite this more confident managing a similar clinical case in the future compared to learners with prior experience of SBT.

## Discussion

This paper presents the evaluation of the SBT-QA10 quality assurance tool. Based on the interview study from which they were derived, [16] we predicted that the ten questionnaire items would provide more specific and useful information for scenario design and debriefing facilitation than overall satisfaction scores. Learner reported perceptions could then guide potentially modifiable influencing factors such as instructor skills and facilitation behaviors [16] and more nuanced management of learners according to their active as opposed to observer roles.

Examining learners’ perceptions to SBT is not uncommon and have been described in previous publication. [15, 31–37] Our study builds on this knowledge by focusing on perceptions that stem from the active phase of the simulation: Apart from “mental effort”, which we recommend is removed, all the items in the SBT-QA10 showed fair correlation suggesting they are all measuring elements of a participant’s experience in the active phase of simulation scenarios. Factor analysis supported these findings and the conclusion from the earlier interview study that learners’ perceptions include both psychosocial and cognitive themes. We proposed that Factor 1 relates to the theme of social connection and support (F1-S), Factor 2 relates to orientation to task (F2-Or), Factor 3 relates to realism (F3-R), and Factor 4 is aligned with mental workload (F4-MWL). To improve the utility of the tool, we recommend that each questionnaire item is denoted by its factor theme, as nominated above. Users should decide whether or not to convert negatively worded statements to positive statements. We believe alternating positive and negative statements strengthens the tool’s validity by reducing opportunities for responders to take cognitive short cuts. While we believe that the best occasion for administering the questionnaire us immediately following the conclusion of the action phase of the scenario, we similarly feel that users are best placed to consider important contextual factors that will determine where the tool best fits into their courses.

The SBT-QA10 enables learners’ perceptions to be deconstructed and quantified, to an extent. This information provides instructors with practical feedback that subsequently can be acted upon to improve their facilitation skills, including pre-scenario briefing, in-scenario debriefing and or rescue strategies. Instructional designers could potentially use the tool to guide elements such as scenario design, the use of remote facilitation technologies and or the separation of active versus observer learners. Course learners may also potentially use the tool to reflect upon their own experience during the debrief and to guide their engagement in future SBT. The tool also has potential utility in research.

Conscious mental effort was one of the eight perceptions in the original interview study [16] where it was defined by the authors as “Low awareness of effort required to interpret a situation” and thought of as impacting our cognition, and thereby affecting the intrinsic cognitive workload. In this study the item for low mental effort did not correlate with a positive experience suggesting it should be omitted. It is well established that cognitive workload impacts upon learning and sources of cognitive workload that are extrinsic and distracting from the learning task should be minimized. [38] Omitting this item from the SBT-QA10 does not preclude any monitoring of a participant’s cognitive workload, since it is indirectly measured in other items including the ability to act independently, feeling supported, scenario realism, understanding the purpose of the scenario and feeling distracted. Other established self-reported measures of cognitive workload are in common use, including the Cognitive Appraisal Index [8] and the single item Paas scale. [39] While we could potentially add these to our monitoring inventory our findings underscore the difficulty of measuring and interpreting cognitive workload, in context.

An unexpected finding of the study was the poor correlation between the ten perceptions and the two measures of satisfaction. As previously mentioned, the questionnaire items were reproduced from an earlier questionnaire-based study involving the DETECT course. [16] Here the measure of comfort was a statistically significant discriminator of face to face versus remote facilitation formats and the measure of confidence was a significant discriminator of instructor experience. Support for these measures was further enhanced in the subsequent interview study from which the perceptions were derived. For example, learners frequently volunteered phrasing describing comfort or enjoyment such as “I felt comfortable/uncomfortable” and “I enjoyed it/did not enjoy it”. Learners also volunteered or responded to probing about the effectiveness of the activity in an affirmative manner using phrasing such as “I learned a lot” or “It was beneficial”. These findings call us to reflect on the meaning of satisfaction scores to program evaluation and reinforce the limitations of relying upon reductionist and quantitative methods to evaluate the impact of learning and its acceptance by learners.

The significant differences between allocated roles found in the SBT-QA10-TPS provide further support for the use of the tool and are consistent with findings reported elsewhere that learners’ experience of simulation are impacted differently by these roles. [18, 19, 40] In this respect, the tool could provide utility to simulation providers to ensure their instructional methods are optimized for both active and observer roles in their training programs. For example, being an active observer who is focused on specific learning outcomes and knowing what to look for has been associated with improved learning in the role. [18, 19, 40] Effective facilitation of observers requires faculty to spend time considering the pre-briefing requirements of this role including setting expectations for active engagement when in the observer role and directly including and specifically asking for the observers’ perspective in the discussion or debrief. [18] Applying these principles is an indication of the value the facilitator places upon the observer role for the purpose of increasing learners’ sense of feeling valued and valuing the observer role. We actively applied these principles as we were aware that some learning outcomes may be better for learners actively engaged in scenarios. [20] Despite this, learners in this study reported higher scores for *feeling part of the team* and *interacting with faculty* when in an active role. These differences were relatively small which may in part reflect a partial closing of an otherwise larger gap between these roles however room for further improvement by faculty appears to be warranted. Other differences represent opportunities by which the observer role may be shown to add value. For instance, as expected, observers scored mental effort lower than active learners. We feel the reduced requirement of observers to attend to tasks provides them the space to look at the bigger picture and to notice details and nuances. [41]

This study uses a relatively narrow interpretation of construct validity considering the multiple dimensions with which contemporary validity frameworks have been adapted in the literature. [24, 38–40] We applied *Relationships with other scores* from Messick’s argument-based validity framework for its direct relevance to the intended use of the SBT-Q10 as a quality assurance tool and avoided other measures of validity evidence as we were confident that they could not be applied. [17, 42]

We intentionally did not introduce additional measures of construct validity in this study due to concerns that major changes in the course format would impact negatively on the courses’ relevance to and suitability for the learners. We note opportunities to further validate the tool through additional measures of construct validity, such as altering any of the scenarios as a means of loading them according to different perceptions as this Similarly, opportunities exist to measure concurrent validity by applying the tool to a course involving high complex scenarios and or to test the generalizability of SBT-QA10 by investigating the learner’s perception from other courses.

We were unable to randomize subjects into groups to minimize between group bias as course logistics prevented this. However, by using a single sample with repeated measures methodology, we felt randomization would have minimal effect on the results, since the learners were exposed to different scenarios, in different roles, with colleagues with different clinical and SBT experience. Nevertheless, the internal consistency of the majority of questionnaire items supports the confirmation process that SBT-QA10 can be used to measure perceptions that impact upon learners’ experiences during DETECT and these findings are strengthened by the significant difference in responses when comparing the observer and active roles which are in concordance with findings in other settings. [20]

We believe the tool has potential utility in further research and could potentially serve as inspiration for others.

## Conclusion

The SBT-QA10 enables learners to convey the different perceptions they experience during simulation and prompts simulation educators and providers to remain aware of the different perceptions learners may have in response to the same event. “With the exception of mental effort, high scores in the other questionnaire items reflects a positive experience for learners during the action phase of SBT and may also influence their experience in subsequent learning activities and the transfer of learning to daily clinical life.” Their inconsistent correlation of the SBT-Q10 items with measures of comfort and confidence raises questions about the determinants of the latter and or their utility as measures of satisfaction. Further research is needed to demonstrate the validity and applicability of the tool in other simulation-based training settings.

In conclusion, we feel the SBT-Q10 has potential utility for course learners who may use the tool to reflect upon their own experience and to guide their engagement in future SBT. The tool may provide instructors with practical feedback that can be acted upon to improve their facilitation skills, including pre-scenario briefing, in-scenario debriefing, and/or rescue strategies. Finally, instructional designers could potentially use the tool to guide elements such as scenario design, the use of technologies and the separation of active versus observer learners.

We believe SBT-QA10 could be used as a simulation “thermometer” i.e., an intervention tool: Do we understand, what we are exposing the learners to? How do they perceive the scenarios? Do they understand the purpose, the content and context of the scenario? Does simulation “always” work? Sometimes it does not and why is that? The learners’ perceptions matter and should be afforded attention. [15, 31–37]

## Electronic supplementary material

Below is the link to the electronic supplementary material.

Supplementary Material 1

## Data Availability

The data supporting the conclusions of this article are included within the article. Corresponding author may be contacted to forward requests for data sharing.

## List of abbreviation

DETECT: Detection, Evaluation, Treatment, Escalation and Communication in Teams
IQR: Inter Quartile Range
*n*: Number
NA: Not Answered
SBT: Simulation-Based Training
SBT-QA10: Simulation Based Training Quality Assurance Tool (including 10 items)
TPS: Total Perception Score
Q1, Q2: Questionnaire item 1, Questionnaire item 2
