# Supplementary material for: Evaluation of the simulation based training quality assurance tool (SBT-QA10) as a measure of learners’ perceptions during the action phase of simulation
Source: BMC Med Educ. 2023 May 1;23:290. doi: 10.1186/s12909-023-04273-6 (PMC10152736; doi:10.1186/s12909-023-04273-6)
Supplement: Supplementary file 1 — Supplementary Material 1 [file 12909_2023_4273_MOESM1_ESM.docx]

**Reprints**

Leonie Watterson, MBBS, FANZCA, MClinEd, GradCertOrgCoach&Lship, Sydney Clinical Skills and Simulation Centre, Level 6, Kolling Research & Education Building, Royal North Shore Hospital, Reserve Rd, St Leonards, NSW 2065, Australia (e‐mail: Leonie@leoniewatterson.com; [Leonie.Watterson@health.nsw.gov.au](mailto:Leonie.Watterson@health.nsw.gov.au)).
